# In-Home Sleep Recordings in Military Veterans With Posttraumatic Stress Disorder Reveal Less REM and Deep Sleep <1 Hz

**DOI:** 10.3389/fnhum.2018.00196

**Published:** 2018-05-11

**Authors:** Julie A. Onton, Scott C. Matthews, Dae Y. Kang, Todd P. Coleman

**Affiliations:** ^1^Institute for Neural Computation, University of California, San Diego, La Jolla, CA, United States; ^2^Warfighter Performance, Naval Health Research Center, San Diego, CA, United States; ^3^Psychiatry, VA San Diego Healthcare System, San Diego, CA, United States; ^4^Department of Psychiatry, University of California, San Diego, La Jolla, CA, United States; ^5^Department of Bioengineering, University of California, San Diego, La Jolla, CA, United States

**Keywords:** EEG, PTSD, sleep, electrodermal activity, slow wave sleep, Lo Deep, REM, sleep scoring algorithm

## Abstract

Veterans with posttraumatic stress disorder (PTSD) often report suboptimal sleep quality, often described as lack of restfulness for unknown reasons. These experiences are sometimes difficult to objectively quantify in sleep lab assessments. Here, we used a streamlined sleep assessment tool to record in-home 2-channel electroencephalogram (EEG) with concurrent collection of electrodermal activity (EDA) and acceleration. Data from a single forehead channel were transformed into a whole-night spectrogram, and sleep stages were classified using a fully automated algorithm. For this study, 71 control subjects and 60 military-related PTSD subjects were analyzed for percentage of time spent in Light, Hi Deep (1–3 Hz), Lo Deep (<1 Hz), and rapid eye movement (REM) sleep stages, as well as sleep efficiency and fragmentation. The results showed a significant tendency for PTSD sleepers to spend a smaller percentage of the night in REM (*p* < 0.0001) and Lo Deep (*p* = 0.001) sleep, while spending a larger percentage of the night in Hi Deep (*p* < 0.0001) sleep. The percentage of combined Hi+Lo Deep sleep did not differ between groups. All sleepers usually showed EDA peaks during Lo, but not Hi, Deep sleep; however, PTSD sleepers were more likely to lack EDA peaks altogether, which usually coincided with a lack of Lo Deep sleep. Linear regressions with all subjects showed that a decreased percentage of REM sleep in PTSD sleepers was accounted for by age, prazosin, SSRIs and SNRIs (*p* < 0.02), while decreased Lo Deep and increased Hi Deep in the PTSD group could not be accounted for by any factor in this study (*p* < 0.005). Linear regression models with only the PTSD group showed that decreased REM correlated with self-reported depression, as measured with the Depression, Anxiety, and Stress Scales (DASS; *p* < 0.00001). DASS anxiety was associated with increased REM time (*p* < 0.0001). This study shows altered sleep patterns in sleepers with PTSD that can be partially accounted for by age and medication use; however, differences in deep sleep related to PTSD could not be linked to any known factor. With several medications [prazosin, selective serotonin reuptake inhibitors (SSRIs), serotonin-norepinephrine reuptake inhibitors (SNRIs); *p* < 0.03], as well as SSRIs were associated with less sleep efficiency (b = -3.3 ± 0.95; *p* = 0.0005) and more sleep fragmentation (b = -1.7 ± 0.51; *p* = 0.0009). Anti-psychotics were associated with less sleep efficiency (b = -4.9 ± 1.4; *p* = 0.0004). Sleep efficiency was negatively impacted by SSRIs, antipsychotic medications, and depression (*p* < 0.008). Increased sleep fragmentation was associated with SSRIs, SNRIs, and anxiety (*p* < 0.009), while prazosin and antipsychotic medications correlated with decreased sleep fragmentation (*p* < 0.05).

## Introduction

Assessing sleep architecture in subjects with posttraumatic stress disorder (PTSD) has produced conflicting results (Kobayashi et al., 2007). The reasons for this could be a number of factors, for example, age of the population, time since trauma, concurrent mental or physical disorders, type of trauma, medication use, quantification methods, and confounding effects of sleeping in a laboratory setting. However, subjective reports from individuals suffering from PTSD are overwhelmingly negative, citing difficulties falling asleep, staying asleep, and low sleep quality even when sleeping through the night (Woodward et al., 1996).

Full sleep laboratory assessment is currently the only way for patients and clinicians to gain objective information about sleep architecture (Capaldi et al., 2011), but the prohibitive cost for these assessments limits access. Typical sleep lab measurements include electroencephalographic (EEG), heart rate, muscle activity, and eye movement data, among other measures (Rechtschaffen and Kales, 1968). However, the final sleep report does not show the actual EEG activity in either raw or spectral format, leaving out potentially important information about brain activity that could be useful for clinician assessments and patient satisfaction (Shrivastava et al., 2014).

Repeated clinical sleep assessments are especially rare, making it difficult to know how particular pharmacological or behavioral interventions affect sleep architecture. A low-cost alternative to a full sleep lab assessment would allow for sleep assessments to be conducted at patient intake, throughout treatment, and following stabilization. This scenario would give both patients and clinicians more objective information about how sleep quality was affected by the chosen intervention.

Traditional sleep scoring guidelines identify deep, or slow wave sleep, based on the presence of EEG activity below 3 Hz. Perhaps because sleep scoring was originally conducted by eye, and efforts to maintain standardization have resisted changing this, it has not been widely accepted or even known that humans express two different modes of slow wave sleep, as has been reported in animal research (Steriade et al., 1993). A recent publication showed that, using simple spectral decomposition, slow oscillations (<1 Hz) and delta (1–3 Hz) were largely expressed in separate sleep cycles and that only the slow oscillations (coined “Lo Deep”) were associated with electrodermal activity (EDA) (Onton et al., 2016). It is not known what role EDA has during sleep, but because of its dramatic rise during Lo Deep sleep, which is even higher than waking levels (Johnson and Lubin, 1966; Hori et al., 1970), it is a useful marker of healthy Lo Deep sleep that can aid clinical assessment of sleep pathology.

Electrodermal activity is usually considered to be a marker of sympathetic nervous system activation in the waking state because it is often triggered by emotional stimuli (Sequeira et al., 2009). The purpose or function of EDA during sleep is unclear. EDA level has been consistently observed to increase during slow wave sleep and decrease during REM in healthy sleepers (Hori et al., 1970). Accordingly, EDA peaks are typically associated with the first half of the night, when slow wave sleep dominates (Sano et al., 2014).

For this report, we used a low profile EEG recording device that allowed subjects to sleep in the comfort of their own homes. The data collected were transformed into spectral format and automatically scored using a previously described algorithm (Onton et al., 2016) that is meant to quantify clear spectral patterns in the sleep data and highlight any differences between PTSD and healthy sleepers. Sleep measurements from PTSD and control sleepers were compared and modeled using linear regressions to determine what underlying factors most affected sleep stage durations, sleep efficiency, and sleep fragmentation.

## Materials and Methods

### Participants

The control population participants were recruited from the general San Diego population by online advertisements and word of mouth and resulted in 70 self-reported healthy sleepers (39 males, 31 females; mean age 29.7 ± 7, range 19–48). Data from these subjects have been previously reported (Onton et al., 2016). Sleepers with PTSD were recruited from the ASPIRE Center, the VA San Diego Healthcare System residential recovery treatment program, where 60 PTSD residents (two females; mean age 35.4 ± 6.9, range 24–55) volunteered to participate in the study. Because of the relatively lower number of females in the PTSD treatment facility, the number of females is much larger in the control population. General linear models with only control subjects showed a significant effect of gender on Light sleep [regression coefficient: -2.3 ± 1.0 (less in males), *P* = 0.016; results not shown], so this factor was included in the general linear models comparing PTSD and control sleepers so that any gender effects would be controlled for while assessing group differences.

Control participants, as reported in Onton et al. (2016), were medication-free and reported no history of traumatic brain injury or other brain-related disorders. Caffeine consumption was allowed, but advised to be moderate according to individual caffeine habits. Alcohol was not allowed on recording days. Alcohol consumption was prohibited by program rules at the ASPIRE center, so PTSD subject recordings were alcohol-free.

All participants gave informed consent, answered questionnaires and were instructed on the use of the sleep-recording equipment. Participants completed three nights of sleep recordings within about 2 weeks on nights of their choice and then returned the device to receive compensation for their participation.

This study was approved by the Institutional Review Board of the Naval Health Research Center in San Diego and the VA San Diego Healthcare System Human Research Protections Program. Participants were informed of the study requirements prior to their written consent.

### Equipment

Details regarding the equipment used have been described elsewhere (Onton et al., 2016). In brief, subjects used a small Avatar amplifier (EGI, Eugene, OR, United States) or a 2-channel Cognionics headband (Cognionics, San Diego, CA, United States). EDA, acceleration, heart rate, and temperature were collected using an Empatica wristband (Empatica, Milano, Italy). Both devices used electrode stickers placed at FP1, FP2, left mastoid and right mastoid.

### Procedure

Upon intake into the study, participants completed the PTSD Checklist (PCL-4; VA National Center for PTSD), the Depression, Anxiety and Stress Scales (DASS), and the Pittsburgh Sleep Quality Index (PSQI). For details of sleep device application, please see Onton et al. (2016).

### Data Processing and Visualization

Details of data processing are identical to those previously described (Onton et al., 2016), except that in cases when the difference channel FP1–FP2, or “forehead-forehead,” channel was unavailable because either FP1 or FP2 was corrupted in some way, the other clean FP1 or FP2 was used instead. This selection procedure was necessary for 19 data sets (5 controls and 14 PTSD).

To ensure that measures were reasonably stable across nights for each subject, we performed a correlation analysis between the first and last night for each subject on all the measures used in this report and found significant correlations for all (Light: *r* = 0.2, *p* = 0.01; Hi Deep: *r* = 0.3, *p* = 0.001; Lo Deep: *r* = 0.4, *p* = 0.0001; Hi+Lo Deep: *r* = 0.2, *p* = 0.05; REM: *r* = 0.5, *p* < 0.0001; Wake: *r* = 0.4, *p* < 0.0001; Fragmentation: *r* = 0.5, *p* < 0.0001; Sleep efficiency: *r* = 0.4, *p* < 0.0001). Conversely, we also performed a paired *t*-tests on the same data and found no significant differences between nights (Light: *p* = 0.5; Hi Deep: *p* = 0.9; Lo Deep: *p* = 0.2; Hi+Lo Deep: *p* = 0.1; REM: *p* < 0.1; Wake: *p* < 0.2; Fragmentation: *p* < 1.0; Sleep efficiency: *p* < 0.2).

In brief summary, data were processed using MATLAB (Mathworks, Natick, MA, United States). Each sleep recording was decomposed into frequency power (dB) between 0.1 and 150 Hz for every 0.5-s time step using Morlet wavelets (3 cycles at 0.1 Hz, 30 cycles at 150 Hz and evenly distributed cycles between). Power was converted to dB by for formula 10^∗^log_10_(power) and then the mean power spectrum was calculated and subtracted from the whole night to produce the visual representation and the band power values for the sleep staging algorithm.

### Sleep Scoring Algorithm

Details of the sleep scoring algorithm have been presented elsewhere (Onton et al., 2016). In brief summary, a hidden Markov model algorithm with subsequent estimation-maximization and Viterbi algorithm was constructed to classify each 30-s stretch of EEG data into one of five stages: Wake, rapid eye movement (REM), Light, Hi Deep, and Lo Deep. Baseline-corrected log power in the following frequency bands were averaged and then used as input to the sleep staging algorithm (Wake: 37–47 Hz; REM: 16–30 Hz; Light: 10.5–16 Hz; Hi Deep: 1–3 Hz; Lo Deep: 0.1–1 Hz).

### Statistics

Because PTSD subjects showed a significant skew toward shorter total sleep time (6.6 h for controls vs. 6.0 h for PTSD sleepers, *p* < 0.0001), this report focuses on percentage of time spent in each sleep stage relative to total sleep time of the corresponding night. Significant differences between control and PTSD populations in terms of percentage of time spent in each stage were calculated by individual *t*-tests corrected for multiple comparisons by multiplying each *p*-value by 8 for the total number of tests conducted (Light, Hi Deep, Lo Deep, Hi+Lo Deep sleep, ratio of Lo/Hi Deep sleep, REM, sleep efficiency, sleep fragmentation). In other words, the *p*-values shown have been adjusted, but original *p*-values were < = 0.00625 (corrected to 0.05) to be considered significant. Sleep efficiency was calculated by dividing the total sleep time by total time in bed. Sleep fragmentation was calculated from the number of stage transitions lasting over three 30-s time points divided by total time after sleep onset until the end of the sleep recording. Groups were compared with *t*-tests corrected for multiple comparisons to determine overall group differences in sleep efficiency and fragmentation.

Multiple linear regression was implemented in MATLAB using the *robustfit()* function. *Robustfit()* uses iteratively reweighted least squares with the bisquare weighting function and differs from *regress()* in that outliers do not overly affect the model. Response variables used for separate regressions were percentage of time in each sleep stage, sleep fragmentation, and overall sleep efficiency (replaced percentage of time awake after sleep onset because of nearly identical results).

The first series of models were created to assess the effect of psychological and medication variables on the chosen sleep measures. For these models, controls were excluded to focus on what factors within the pathological population could account for the differences in mean sleep measures. Gender was not included because of the very small number of females (2) in the PTSD group. Age was excluded from these models because it was found to be unrelated to response variables in the PTSD population. PCL-4 scores were excluded from the final model because they were consistently unrelated to any response variable. DASS depression, anxiety, and stress subscale scores were included in the final models, and submitted as three separate subdivisions.

Medications were submitted to the PTSD-only model according to drug class, with the exception of medications that were taken with high enough frequency to be evaluated individually, as was the case for prazosin and trazodone. Not all medications present in the subject population were common enough to be included in any of the following drug classes, and drug interactions were not accounted for in this analysis. Drug classes were as follows: selective serotonin reuptake inhibitors (SSRIs): citalopram, fluoxetine, escitalopram, sertraline; serotonin-norepinephrine reuptake inhibitors (SNRIs): duloxetine, venlafaxine, mood stabilizers/anticonvulsants: carbamazepine, gabapentin, lamotrigine, pregabalin, levetiracetam, valproic acid, topiramate; benzodiazepines: diazepam, clonazepam, lorazepam; opioids: buprenorphine/naloxone, hydrocodone, tramadol; antipsychotics: risperidone, quetiapine, ziprasidone; sedative hypnotics: zolpidem, eszopiclone; antihistamines: hydroxyzine, diphenhydramine; and headache/migraine medicines: valproic acid, topiramate. Sedative hypnotics did not show any significant effect on any response variable tested and were removed from the final model. Mood stabilizers/anticonvulsants and headache/migraine medications had only tiny and therefore meaningless effects on sleep measures (less than 0.001% of the night) and thus were removed from the final model. Benzodiazepines and opioids did not show any significant effects on sleep measure responses, possibly due to the small number of subjects taking them (20 and 16, respectively), or because these medications are frequently taken only as needed and may not have been taken on recording nights. Finally, trazodone and antihistamines were removed from the model due to lack of significant effect on any sleep measure responses. The final medications submitted to the final model were prazosin, SSRIs, SNRIs, and antipsychotic medications.

For the model to determine the effect of group (PTSD or control) on sleep measures, age, gender and the four medication classes found to correlate with sleep measures (prazosin, SSRIs, SNRIs, anti-psychotics) were included to determine whether group differences are still significant after controlling for those factors.

## Results

PSQI scores were significantly higher (worse) for the PTSD group (mean total score 13.4 ± 4.5) compared with control subjects (mean total score of 2.9 ± 1.7; *p* < 0.00001; *t*-test).

**Table 1** shows the percentage of time in each sleep stage for both groups, as well as males only from each group to demonstrate that the gender imbalance does not affect the results. All stages showed a significant difference in the PTSD population, with Light (*p* = 0.03) and Hi Deep (*p* < 0.0001) sleep lasting longer, and REM (*p* < 0.0001) and Lo Deep sleep (*p* = 0.001) shorter, on average, than among control subjects (*t*-test, correcting for multiple comparisons). The only exception was that combined Hi+Lo Deep sleep (total slow wave sleep) did not differ between controls and the PTSD population. Stage measures for only males subjects remained significantly different between groups except combined Hi+Lo Deep sleep, which was similarly non-significantly different (REM, Light, Hi and Lo Deep *p* < = 0.01; *t*-test corrected for multiple comparisons). To test whether, within the same night, Hi or Lo Deep sleep was replaced with other sleep stages or with the other type of deep sleep (Hi or Lo), the log ratio of Lo to Hi Deep sleep for each night was calculated. The results showed that controls had significantly more Lo Deep than Hi Deep sleep (log ratio of Lo/Hi: 0.12) while PTSD subjects usually had more Hi Deep than Lo Deep sleep (log ratio of Lo/Hi: -0.11; *p* = 0.0003, *t*-test, correcting for multiple comparisons). The log ratio of Lo to Hi Deep sleep in males only was very similar to the full groups in both control and PTSD populations (*p* = 0.01, *t*-test, correcting for multiple comparisons). Note that many PTSD nights were in the healthy control range and the significant skews were due to only a portion of nights that fell more heavily in the tails of the control distribution. This distribution allowed for further investigation of the PTSD population alone to determine what factors contributed to the abnormal nights.

**Table 1:** Percentage of night spent in each sleep stage.

	Light	Hi Deep	Lo Deep	Hi+Lo Deep	REM	Lo/Hi Deep Ratio
Controls	25.4 ± 6.7	16.4 ± 7.7	21.7 ± 10.5	38.1 ± 8.3	29.8 ± 7.1	0.12 ± 0.4
PTSD	27.8 ± 8.9*	20.9 ± 10.0*	17.4 ± 10.6*	38.2 ± 8.8	25.8 ± 9.1*	-0.10 ± 0.6*
Male controls	24.4 ± 6.4	16.7 ± 8.0	22.0 ± 10.0	38.6 ± 7.6	29.4 ± 6.6	0.12 ± 0.5
Male PTSD	27.7 ± 9.0*	20.7 ± 10.0*	17.7 ± 10.6*	38.3 ± 8.8	26.0 ± 9.2*	-0.09 ± 0.6*


*Values are means ± standard deviations. ^∗^Significantly different (*p* < 0.05) from control group as determined by *t*-test corrected for multiple comparisons. PTSD, posttraumatic stress disorder; REM, rapid eye movement.*

Sleep efficiency scores for all subjects ranged from 62 to 100%, with the PTSD group having significantly worse sleep efficiency on average (controls: 92.9 ± 5.1; PTSD: 91.0 ± 7.3; *p* < 0.05, *t*-test corrected for multiple comparisons), though the largely overlapping distributions and relatively high average scores suggest that this difference is of minimal clinical importance. Sleep fragmentation scores generally fell between 5 and 20 for all subjects, with two outliers near 29 in the PTSD group. Sleep fragmentation was not significantly different between groups (controls: 9.2 ± 2.9; PTSD: 10.0 ± 3.9). Because of potential gender differences in sleep architecture, these sleep measures were also calculated for only male subjects in each group (39 controls, 58 PTSD). Sleep efficiency for male control subjects was 92.1 ± 5.7 and for male PTSD subjects was 91.2 ± 7.2, which did not reach significance even though the means were relatively similar to males and females. Sleep fragmentation for male control subjects was 9.3 ± 3.1 and for male PTSD subjects was 9.8 ± 2.7, which was also substantially similar to the mixed gender values and also not significantly different.

Linear regressions were calculated to reveal possible correlates of the sleep measures that differed in the PTSD group compared to controls (**Table 2**). The factors evaluated were several medication categories and subjectively reported DASS scores (age and gender were excluded because of lack of effect and small number of subjects, respectively). Medication categories included were prazosin, SSRIs, SNRIs, and antipsychotics (sedative hypnotics, mood stabilizers/anticonvulsants, headache/migraine medicines, benzodiazepines, opioids, trazodone, and antihistamines were eliminated from the final models because they did not show any significant associations with any sleep measure). The outcome variables were percentage of time in each sleep stage, sleep efficiency, and sleep fragmentation. Prazosin was associated with a significant decrease in REM proportion (b = -5.9 ± 1.6; *p* = 0.0003) and an increase in Hi+Lo Deep sleep (b = 3.5 ± 1.7; *p* = 0.041), as well as a slight decrease in sleep fragmentation (b = -1.3 ± 0.6, *p* = 0.04). SSRIs were associated with less REM (b = -4.5 ± 1.5; *p* = 0.004), lowered sleep efficiency (b = -4.2 ± 1.1; *p* = 0.0001), and increased sleep fragmentation (b = 2.0 ± 0.6; *p* = 0.001). SNRIs also significantly decreased REM (b = -4.0 ± 1.8; *p* = 0.027) and increased sleep fragmentation (1.9 ± 0.7; *p* = 0.006), but did not have any effect on sleep efficiency. Antipsychotics did not affect any single sleep stage, but they were associated with significantly lowered sleep efficiency (b = -5.9 ± 1.5; *p* = 0.0002) and decreased sleep fragmentation (b = -1.7 ± 0.8; *p* = 0.05). Self-reported DASS depression was associated with a significant decrease in REM sleep (b = -6.2 ± 1.3; *p* = 0.000006), as well as a decrease in sleep efficiency (b = -2.4 ± 0.9; *p* = 0.008). Conversely, DASS anxiety was associated with increased REM sleep (b = 6.4 ± 1.6; *p* = 0.00007), as well as increased sleep fragmentation (b = 1.6 ± 0.6; *p* = 0.009). Finally, DASS stress symptoms correlated with decreased Hi Deep (b = -4.7 ± 1.3; *p* = 0.001) and increased Lo Deep sleep (b = 5.2 ± 1.4; *p* = 0.0002). The number of outliers is included in **Table 2** because it gives a metric for the ability of the model to include all available data, and also shows whether observed effects might affect one group more than the other. Percent outliers were <8% for all regressions in this table.

**Table 2:** Results of PTSD-only linear regression models with medications and psychological measures.

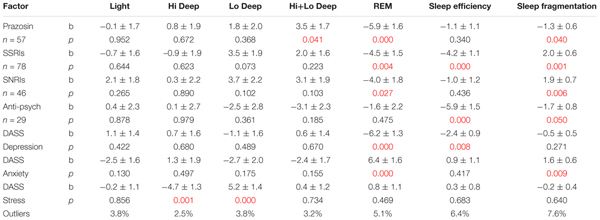


*b, regression coefficient; *p*, significance level; b-values are mean ± standard error; red font indicates significance of *p* < 0.05. Each column is outcome variable of independent models. Number below each medication indicates number of subjects taking the respective medication. DASS, Depression Anxiety Stress Scales; PTSD, posttraumatic stress disorder; REM, rapid eye movement; SNRIs, serotonin-norepinephrine reuptake inhibitors; SSRIs, selective serotonin reuptake inhibitors.*

The medications that were found to correlate to one or more sleep measures were then included in a series of regressions to determine group differences while controlling for medication, age and gender (**Table 3**). These regressions show a significant effect of gender on Light sleep (b = -2.4 ± 1.1; *p* = 0.02), showing slightly less in males than in females. Age showed a significant correlation with percentage of REM sleep (b = -0.15 ± 0.06; *p* = 0.02), with less REM in older subjects. The medication correlations very much echoed the findings from the previous set of regressions in just PTSD population alone. Prazosin correlated with more Hi+Lo Deep sleep (b = -3.3 ± 1.6; *p* = 0.04) and less REM (b = -4.9 ± 1.4; *p* = 0.0006). SSRIs were associated with less REM (b = -4.1 ± 1.4; *p* = 0.003), less sleep efficiency (b = -3.3 ± 0.95; *p* = 0.0005) and more sleep fragmentation (b = -1.7 ± 0.51; *p* = 0.0009). SNRIs correlated with less REM, as before (b = -5.5 ± 1.6; *p* = 0.0007), but also became significantly related to more Hi+Lo Deep sleep (b = -3.7 ± 1.8; *p* = 0.04). Finally, anti-psychotics remained associated with less sleep efficiency (b = -4.9 ± 1.4; *p* = 0.0004), but not with more sleep fragmentation as before. With these factors controlled, group differences between PTSD and control subjects were significant in both Hi and Lo Deep sleep measurements, but not for Hi+Lo Deep sleep (i.e., all Deep sleep). Hi Deep sleep generally subsumed a larger percentage of the night in PTSD subjects (b = -4.4 ± 1.5; *p* = 0.005) and Lo Deep sleep was far less prevalent in PTSD subjects (b = -8.1 ± 1.9; *p* = 0.00003). Differences in REM sleep measurements were not significant between groups when age and medications were controlled for. Percentage of outliers varied between 1.6% in the control group for Lo Deep sleep and 10.6% in the PTSD group for sleep efficiency.

**Table 3:** Results of control vs. PTSD linear regression models with age, gender and medications accounted for.

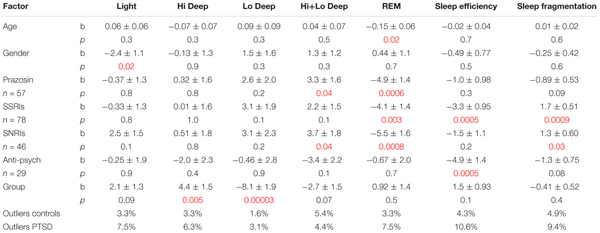


*b, regression coefficient; *p*, significance level; b-values are mean ± standard error; red font indicates significance of *p* < 0.05. Each column is outcome variable of independent models. Number below each medication indicates number of subjects taking the respective medication. DASS, Depression Anxiety Stress Scales; PTSD, posttraumatic stress disorder; REM, rapid eye movement; SNRIs, serotonin-norepinephrine reuptake inhibitors; SSRIs, selective serotonin reuptake inhibitors.*

**FIGURE 1:**
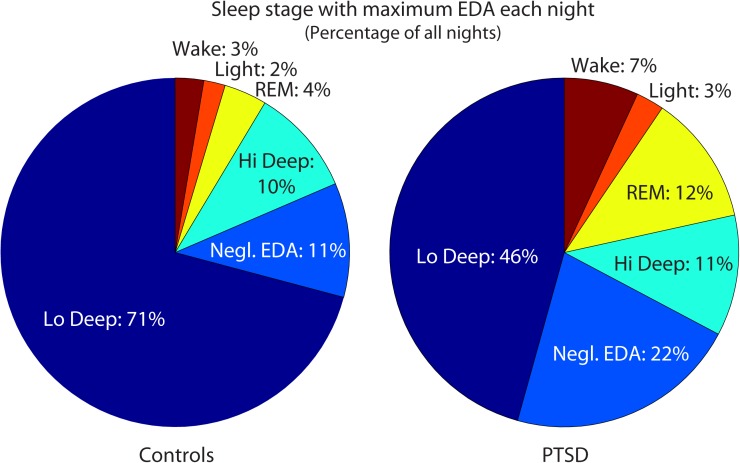
Maximum EDA magnitudes were most common during Lo Deep sleep in both groups. However, relatively more PTSD-sleeper nights had maximum EDA magnitude during REM sleep or no appreciable changes in EDA level. EDA, electrodermal activity; Negl., negligible; PTSD, posttraumatic stress disorder; REM, rapid eye movement.

Posttraumatic stress disorder sleepers also tended to differ from control subjects in their EDA. Control subjects showed maximal EDA magnitude during Lo Deep sleep for 71% of the total nights recorded (182 control and 157 PTSD nights with EDA). In contrast, PTSD sleepers showed maximal EDA during Lo Deep 46% of nights, and instead sometimes showed maximal EDA during REM (12%) or did not show appreciable EDA shifts at all during the night (22%, **Figure 1**). The average maximum EDA magnitude (±SD) in control sleepers during Lo Deep was 4.9 ± 5.6 (range: 0.01 to 32.6). PTSD sleeper EDA maximum was not significantly different at 3.8 ± 5.2 (range: -0.05 to 29.6). During REM, the average maximum control EDA was 2.7 ± 3.4 (range: -0.07 to 15.0), which was not significantly different from PTSD sleepers who had a maximum EDA of 2.5 ± 4.2 (range: 0.0007 to 24.0).

**FIGURE 2:**
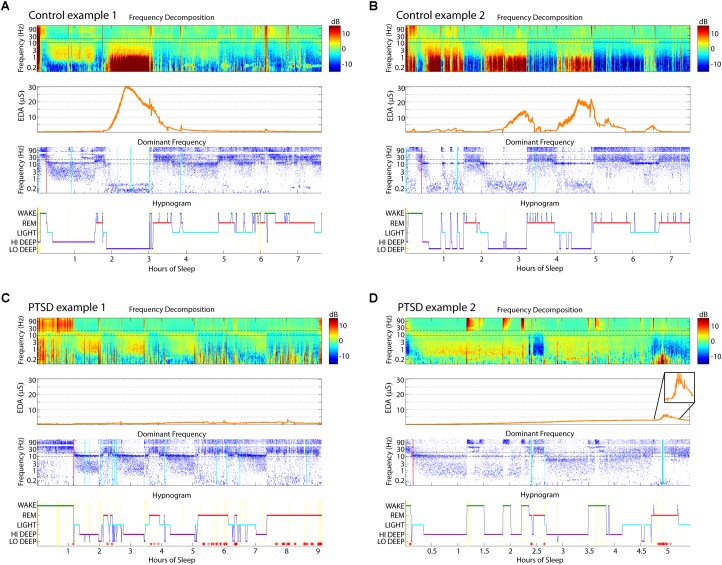
Sleep report examples from two control **(A,B)** and two PTSD **(C,D)** sleepers. These control sleepers showed Lo Deep sleep with associated EDA, while the PTSD sleepers showed a lack of Lo Deep and associated EDA. **(A)** Hi Deep was not associated with EDA peaks. **(B)** EDA can have variable magnitude during different cycles of Lo Deep sleep. **(C)** PTSD sleepers more often showed no substantial changes at all, or **(D)** increases during REM. Inset in **(D)** shows the EDA activity at higher magnification to highlight the signal characteristics. Red asterisks on hypnogram indicate post-algorithm adjustment from Lo Deep to REM when 25 Hz band is higher power than spindle band. Cyan lines on the Dominant Frequency panel indicate moments of large EEG deflections likely due to movement. Red lines on the Dominant Frequency panel indicate the estimated time of sleep onset. EDA, electrodermal activity; EEG, electroencephalography; PTSD, posttraumatic stress disorder, REM, rapid eye movement.

Example sleep reports from healthy and PTSD sleepers are shown in **Figure 2** to illustrate the typical EDA magnitude and correspondence with Lo Deep in control subjects (**Figures 2A,B**), as well as variations in EDA pattern in some PTSD sleepers (**Figures 2C,D**). **Figure 2A** shows a cycle of Hi Deep sleep during the first 90 min, which did not have an associated EDA increase, followed by a cycle of Lo Deep sleep, which did have a simultaneous increase in EDA to well above 20 μS. EDA began to decrease at the offset of Lo Deep sleep and did not increase appreciably the rest of the night, except for a small rise during what appears to be a brief awakening after hour 6 (**Figure 2A**). **Figure 2B** shows another control subject who did not show any Hi Deep sleep, but rather several cycles of Lo Deep sleep that were all associated with various levels of EDA, up to a maximum magnitude of about 20 μS. While many PTSD sleepers also showed this association between Lo Deep and EDA, **Figure 2** shows two examples of how some PTSD sleepers showed negligible EDA throughout the night or increased EDA during REM when the EDA signature differed qualitatively from increases during Lo Deep sleep. **Figure 2C** shows an example of a PTSD sleeper whose EDA never left background levels and showed no clear pattern across cycles. This subject, as well as the PTSD sleeper in **Figure 2D**, showed no Lo Deep sleep. Instead, these sleepers expressed mostly Hi Deep sleep, with varying amounts of REM and Light sleep. **Figure 2D** shows an example of EDA response during REM sleep. This period can be fairly confidently categorized as REM because of the clear absence of sleep spindles during that stretch. Note the EDA scales for both PTSD plots are much smaller than for the control-sleeper panels, which also suggests abnormal overnight EDA magnitude in these PTSD subjects.

**FIGURE 3:**
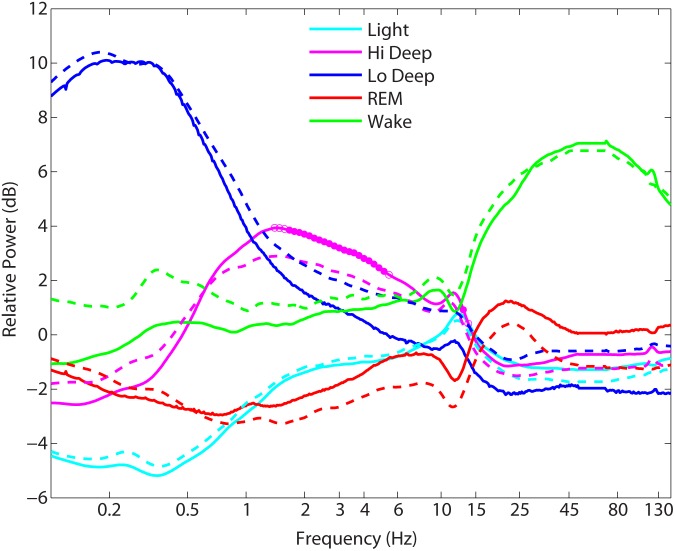
Mean power spectra during each sleep stage across each group. Solid traces = controls, dashed traces = posttraumatic stress disorder (PTSD) sleepers. The only significant differences between controls and PTSD sleepers ranged between 1.3–5.4 Hz and 12.2–13.8 Hz in Hi Deep sleep, where PTSD sleepers showed significantly lower relative power than control sleepers (*p* < 0.003).

The relative power spectra for each stage were compared across groups to quantify any differences in spectral power during the various sleep stages. **Figure 3** shows the stark differences between stages in the relative power spectra (mean across entire night removed). Note the dip in spindle frequency (∼12–13 Hz) in the REM traces compared with the peaks in non-REM sleep. Wake also has a dip in spindle frequencies, but shows instead a peak in the alpha range (∼10 Hz). However, group differences in spectral power were minimal, with the exception of power in the 1.6–5.1 Hz range and at 13 Hz during Hi Deep sleep (solid circles: *p* < 0.001, by ANOVA after Bonferroni correction), where PTSD sleepers showed significantly lower relative power (**Figure 3**). At a *p*-value of 0.003, the ranges were 1.3–5.4 Hz and 12.2–13.8 Hz (open circles: 0.001 < *p* < 0.003, by ANOVA after Bonferroni correction). Whole-night raw spectra were compared between groups for each spectral range used for the sleep scoring algorithm and no significant differences were found (*p* < 0.1).

## Discussion

The results from this study show, both by *t*-test comparisons and linear regression models that PTSD sleepers tended to fall outside control sleeper ranges in several sleep measures. PTSD distributions were highly overlapping with control sleepers, indicating the PTSD population was not uniformly abnormal in their sleep patterns. This is consistent with the PTSD sleep literature, but it also makes intuitive sense given the wide range of symptoms that can occur under the same diagnosis of PTSD. Some studies have found no statistically significant differences in sleep measures (Hurwitz et al., 1998), while others have found abnormalities related to specific symptoms of PTSD (Mellman et al., 1995; Woodward et al., 2000). A meta-analysis across 20 studies of PTSD patients found significant effect sizes for increased stage 1, decreased slow wave sleep, and increased REM density during REM sleep (Kobayashi et al., 2007). The same study also found that moderating factors, such as age, gender, depression, and substance use, had significant effects on the results (Kobayashi et al., 2007).

Pittsburgh Sleep Quality Index scores collected for this study were very similar to values in the literature. Control subjects in our study had an average score of 2.9 ± 1.7, which is in the range of control subjects in the first validation study who had an average score of 2.7 ± 1.7 (Buysse et al., 1989). Our PTSD group had a mean total score of 13.4 ± 4.5, compared with 14.2 ± 3.3 from a published report using a similar military PTSD population (Ulmer et al., 2011).

Importantly, the sleep stages discussed in the present report do not map directly on to traditional sleep stages because they were determined by spectral rather than time-domain features. REM sleep identification appears to be consistent with traditional visual scoring for control subjects because the amount and percentage of REM sleep were fairly consistent with previously reported values in the literature (29.8% vs. 20–25%) (Carskadon and Dement, 2011). However, due to the differences in scoring, the proportions of Light (25.4%) and Hi+Lo Deep (38.1%) sleep in this study tend to differ from proportions of stage 2 (∼45–55%) and stage 3 (∼13%–23%) reported in the literature (Carskadon and Dement, 2011; Onton et al., 2016); in fact, they appear to be reversed in amount. The reason is likely that the current algorithm scores non-REM sleep as slow wave sleep any time the relative power below 3 Hz is higher than that of spindles (∼12–15 Hz). This means that a 30-s epoch might contain spindle activity throughout, but the average power below 3 Hz may be higher and thus scored as Hi or Lo Deep sleep instead of Light. In traditional scoring rules, if the activity below 3 Hz occurred in less than 20% of the epoch, then it would be scored as stage 2, regardless of the average amplitude during the epoch. Given the reversal of proportions of Light/stage 2 and Hi+Lo Deep/stage 3 in the current method compared with traditional scoring, it would appear it is fairly common for average power below 3 Hz to exceed spindle power while only subsuming 20% of the epoch. Although the scoring method used in this paper is a completely different approach to sleep scoring, this does not automatically invalidate the findings. This scoring clearly follows easily observable patterns in the time-frequency spectral display and is just as likely to correspond to global shifts in brain activity as the traditional method of sleep scoring (Onton et al., 2016).

The present study found a tendency for some PTSD subjects to express less Lo Deep sleep and more Hi Deep sleep, while the sum of Hi and Lo Deep sleep indicated that total slow wave sleep was generally conserved within subject. Some studies have reported PTSD-related abnormalities in deep sleep, usually reporting less than control subjects (Glaubman et al., 1990). Indeed, a meta-analysis across 20 studies suggests the most consistent result for PTSD sleepers was a decrease in slow wave sleep. Given that the current algorithm was probably more likely to categorize stage 2/Light as Hi Deep sleep, it is possible that traditional scoring would only score Lo Deep (21.7% for control subjects) as stage 3 (∼13%–23% in literature) sleep. Indeed, PTSD sleepers in the current study did show significantly less Lo Deep sleep, in line with previous reports of less slow wave sleep. The relatively new categorization of Hi Deep sleep seems physiologically plausible for two reasons. First, without it, some sleepers would have no deep sleep, which is not physiologically plausible, especially given the overall structure of the spectrogram in these cases. And second, in combination with Lo Deep sleep, the total proportion of slow wave sleep is similar to that of control subjects, which would be in line with the idea that deep sleep is generally kept in homeostasis over successive sleep periods (Borbely et al., 1981). Thus, it appears from the present results that PTSD patients simply have a difference in slow wave frequency (Hi vs. Lo) rather than a shift in total amount of deep sleep. If this is the case, Hi Deep should be sufficient to reset, at least partially, the adenosine buildup in the basal forebrain that is known to increase sleep pressure (Borb and Achermann, 1999). Nevertheless, because Lo but not Hi Deep sleep is associated with strong EDA peaks and likely other factors (Onton et al., 2016), Hi and Lo Deep sleep should not be considered equivalent in physiological function. Thus, the present report indicates a possible target for sleep therapy that may have been overlooked in past studies because of limitations of visual scoring in the time domain.

The shift to more Hi Deep and less Lo Deep sleep did not correlate with use of any medication category tested in the regression models. The only predictor that affected the Hi/Lo Deep balance was subjectively reported DASS stress which actually indicated that more stress led to *more* Lo Deep and *less* Hi Deep (whereas PTSD subjects as a whole had less Lo Deep sleep than did control subjects). Thus, it is still unknown what factor within our PTSD group led to less Lo Deep sleep in some cases.

Sleep efficiency and sleep fragmentation were not statistically different on a group level between PTSD and control populations. However, every predictor except DASS stress in the PTSD models indicated a significant influence on one or the other. Sleep efficiency (similar to wake after sleep onset except it adds the time to sleep onset), was not significantly correlated with PTSD symptoms in a published meta-analysis, regardless of comorbid depression or substance use disorder (Kobayashi et al., 2007). However, in that analysis, medication use was not consistent or accounted for, and depression was assessed by a simple high/low split. Thus, high variability would be expected in their calculations, perhaps obscuring associations between sleep measures and underlying factors. In our regressions looking for group differences, sleep efficiency and fragmentation were associated with anti-psychotic medications, SSRIs and SNRIs; and when those were controlled for, there were no group differences in these measures. This suggests that sleep efficiency and fragmentation may be partially related to pharmacological factors, as well as psychological factors such as depression and anxiety.

Regression models using predictors thought to influence sleep measures in the PTSD population identified several drug classes that significantly correlated with one or more sleep measure. SSRIs had the largest effect on sleep measures, affecting REM, sleep efficiency, and sleep fragmentation, which is in line with the widely observed REM suppression in both humans (Sharpley et al., 1996) and animals (Gervasoni et al., 2002). Similarly, SNRIs, which also decreased REM and increased fragmentation in the present study, are documented to cause REM suppression in healthy and depressed people (Salin-Pascual et al., 1997; Chalon et al., 2005; Kluge et al., 2007), as well as in rats (Salin-Pascual and Moro-Lopez, 1997; Sanchez et al., 2007).

Prazosin has been shown to improve nightmares and other PTSD symptoms in both combat and civilian patients with PTSD (Taylor and Raskind, 2002; Peskind et al., 2003; Raskind et al., 2007). One study of Prazosin’s effects on sleep architecture was with civilian trauma patients, which showed increased sleep time, and, inconsistent with our results, increased REM time (Taylor et al., 2008). Studies with rats and cats do not help to resolve the discrepancy as they show both increases and decreases in REM sleep time (Hilakivi et al., 1980; Hilakivi and Leppavuori, 1984; Pellejero et al., 1984; Makela and Hilakivi, 1986; Kleinlogel, 1989). Thus, the effect of prazosin, specifically in the combat veteran PTSD population, requires more research to definitively determine sleep architecture effects of the drug. Given the current results, it is possible that prazosin decreases nightmares by decreasing REM time, which could have a detrimental effect on overall recovery (Lamarche and De Koninck, 2007).

Studies of antipsychotic medication (risperidone, quetiapine, ziprasidone), which predicted lowered sleep efficiency in the present study, have shown mixed results. While in some instances sleep efficiency was reported to improved (Cohrs et al., 2005; Baskaran et al., 2013; Monti et al., 2017), several studies indicate possible sleep disruptions (Keshavan et al., 2007; Gedge et al., 2010; Monti et al., 2017), which is in line with the present findings of decreased sleep efficiency caused by antipsychotic medications in the PTSD population.

Our findings showed less EDA activation in the PTSD population. The significance of this is not clear as the purpose of EDA during sleep is not known. Perhaps the opening of sweat glands that triggers EDA is to allow for thermoregulation during deep sleep (Carskadon and Dement, 2011), or perhaps it is simply activated along with sleep-related nervous system alterations but does not itself perform a crucial function. While EDA has been observed predominantly during slow wave sleep, one study noted the first full sleep cycle was less likely to show EDA during slow wave sleep than subsequent cycles (Freixa i Baque et al., 1983), perhaps due to the first cycle often containing Hi instead of Lo Deep sleep. It would be of interest to reanalyze such studies using the present differentiation between Hi and Lo Deep sleep to better understand the association.

Previous research on EDA during sleep focused primarily on a phenomenon called “storming,” which refers to rapid fluctuations in the skin potential responses that are defined variably as subsuming greater than 20% of an epoch (Fuller et al., 1994) or five cycles per minute for 10 min (Lester et al., 1967). From studies depicting raw data traces, it appears that storming and EDA magnitude elevations tend to coincide and can likely be considered generally synonymous for this discussion (Sano et al., 2014). Consistent with the current results, EDA storming in chronic PTSD patients has been shown to be less than in controls, and this decrease in EDA coincided with a decreased amount of slow wave sleep (Fuller et al., 1994). Similarly, the same researchers found that high-anxiety subjects without PTSD showed less EDA storming than low-anxiety subjects, in addition to less slow wave sleep (Fuller et al., 1997), thus suggesting that anxiety may be a key factor in the amount of slow wave sleep and attendant EDA. However, in the present report, self-reported anxiety levels did not significantly correlate with the amount of Hi or Lo Deep sleep. While EDA during sleep requires more research to determine the functional relevance and behavioral correlates, it appears from the present study to be a fairly reliable marker of Lo Deep sleep and may, therefore, be a useful adjunct measurement to confirm EEG observations of Lo Deep sleep.

While EDA is generally known to appear predominantly during deep sleep, observations of EDA during REM sleep have also been reported in the literature. In one report, subjects were awakened during REM while EDA and/or eye movements were present. Their results indicated the presence of EDA was associated with “bizarre” mentation compared with reports when EDA was not active (Kushniruk et al., 1985). In the present report, the character of the EDA peak during REM appeared qualitatively different from that during Lo Deep, suggesting a slightly different physiological phenomenon. The Lo Deep EDA was a high amplitude hump (with relatively tiny storming riding on top), whereas EDA during REM tended to consist of a series of chaotic spiky peaks of generally lower magnitude than healthy EDA during Lo Deep sleep. It is likely the EDA signature during REM sleep is actually a sympathetic nervous system response to dream content, similar to the waking response to emotional stimuli. Noting the difference between Lo Deep and REM EDA may assist clinicians in detecting healthy Lo Deep sleep and perhaps in identifying potentially troubling nightmares during REM.

The specific decrease in spectral power reported here during Hi Deep sleep in the ∼1.5–5 Hz range is in line with animal research of slow wave sleep. It has been shown that intracerebral injection of corticotropin-releasing hormone (CRH), a stress-related hormone known to be high in PTSD patients (de Kloet et al., 2008), causes a decrease in both duration and the 1–6 Hz spectral power of slow wave sleep (Ehlers et al., 1986). PTSD is associated with a hyperresponsive CRH system and a chronic low level proinflammatory state (von Kanel et al., 2007), which further drives CRH production (Karalis et al., 1997). While the spectral differences here are relative to the spectral power across the whole night of each subject, we did not find any group differences in whole night spectral power, suggesting that this finding might be a true decrease in delta amplitude during Hi Deep sleep in PTSD subjects.

## Conclusion

The present report suggests that some patients with PTSD are deficient in Lo Deep and/or REM sleep, partially attributable to medication effects, but also to unknown regulators, particularly in the case of Lo Deep sleep. These associations suggest that sleep EEG recorded by a simple 2-channel device can be used effectively in a patient’s home to provide powerful information regarding sleep architecture capable of providing rapid assessments in diverse clinical settings.

## Author Contributions

JO designed the study, conducted data collection, performed data analysis and wrote the manuscript. As a psychiatrist, SM participated in interpretation of the results and manuscript production. DK and TC created the sleep scoring algorithm, helped to optimize its use and participated in manuscript revision.

## Conflict of Interest Statement

The authors declare that the research was conducted in the absence of any commercial or financial relationships that could be construed as a potential conflict of interest.

## References

[B1] Baskaran A. Summers D. Willing S. L. Jokic R. Milev R. (2013). Sleep architecture in ziprasidone-treated bipolar depression: a pilot study. 3 139–149. 10.1177/2045125312467348 24167686 PMC3805453

[B2] Borb A. A. Achermann P. (1999). Sleep homeostasis and models of sleep regulation. 14 559–570. 10.1177/074873099129000894 10643753

[B3] Borbely A. A. Baumann F. Brandeis D. Strauch I. Lehmann D. (1981). Sleep deprivation: effect on sleep stages and eeg power density in man. 51 483–495. 10.1016/0013-4694(81)90225-X 6165548

[B4] Buysse D. J. Reynolds C. F. III. Monk T. H. Berman S. R. Kupfer D. J. (1989). The pittsburgh sleep quality index: a new instrument for psychiatric practice and research. 28 193–213. 10.1016/0165-1781(89)90047-4 2748771

[B5] Capaldi V. F. II. Guerrero M. L. Killgore W. D. (2011). Sleep disruptions among returning combat veterans from Iraq and Afghanistan. 176 879–888. 10.7205/MILMED-D-10-00440 21882777

[B6] Carskadon M. A. Dement W. C. (2011). “Monitoring and staging human sleep,” in , 5th Edn, eds Kryger M. Roth T. Dement W. (New York, NY: Elsevier), 16–26. 10.1016/B978-1-4160-6645-3.00002-5

[B7] Chalon S. Pereira A. Lainey E. Vandenhende F. Watkin J. G. Staner L. (2005). Comparative effects of duloxetine and desipramine on sleep eeg in healthy subjects. 177 357–365. 10.1007/s00213-004-1961-0 15290000

[B8] Cohrs S. Meier A. Neumann A. C. Jordan W. Ruther E. Rodenbeck A. (2005). Improved sleep continuity and increased slow wave sleep and rem latency during ziprasidone treatment: a randomized, controlled, crossover trial of 12 healthy male subjects. 66 989–996. 10.4088/JCP.v66n0805 16086613

[B9] de Kloet C. S. Vermetten E. Geuze E. Lentjes E. G. Heijnen C. J. Stalla G. K. (2008). Elevated plasma corticotrophin-releasing hormone levels in veterans with posttraumatic stress disorder. 167 287–291. 10.1016/S0079-6123(07)67025-3 18037027

[B10] Ehlers C. L. Reed T. K. Henriksen S. J. (1986). Effects of corticotropin-releasing factor and growth hormone-releasing factor on sleep and activity in rats. 42 467–474. 10.1159/000124489 3084988

[B11] Freixa i Baque E. Chevalier B. Grubar J. C. Lambert C. Lancry A. Leconte P. (1983). Spontaneous electrodermal activity during sleep in man: an intranight study. 6 77–81. 10.1093/sleep/6.1.77 6844801

[B12] Fuller K. H. Waters W. F. Binks P. G. Anderson T. (1997). Generalized anxiety and sleep architecture: a polysomnographic investigation. 20 370–376. 10.1093/sleep/20.5.370 9381061

[B13] Fuller K. H. Waters W. F. Scott O. (1994). An investigation of slow-wave sleep processes in chronic ptsd patients. 8 227–236. 10.1016/0887-6185(94)90004-3

[B14] Gedge L. Lazowski L. Murray D. Jokic R. Milev R. (2010). Effects of quetiapine on sleep architecture in patients with unipolar or bipolar depression. 6 501–508. 10.2147/ndt.s12433 PMC2938299 20856913

[B15] Gervasoni D. Panconi E. Henninot V. Boissard R. Barbagli B. Fort P. (2002). Effect of chronic treatment with milnacipran on sleep architecture in rats compared with paroxetine and imipramine. 73 557–563. 10.1016/S0091-3057(02)00812-2 12151030

[B16] Glaubman H. Mikulincer M. Porat A. Wasserman O. Birger M. (1990). Sleep of chronic post-traumatic patients. 3 255–263. 10.1002/jts.2490030207

[B17] Hilakivi I. Leppavuori A. (1984). Effects of methoxamine, and alpha-1 adrenoceptor agonist, and prazosin, an alpha-1 antagonist, on the stages of the sleep-waking cycle in the cat. 120 363–372. 10.1111/j.1748-1716.1984.tb07396.x 6146242

[B18] Hilakivi I. Leppavuori A. Putkonen P. T. (1980). Prazosin increases paradoxical sleep. 65 417–420. 10.1016/0014-2999(80)90346-5 6250858

[B19] Hori T. Miyasita A. Niimi Y. (1970). Skin potential activities and their regional differences during normal sleep in humans. 20 657–671. 10.2170/jjphysiol.20.657 4324224

[B20] Hurwitz T. D. Mahowald M. W. Kuskowski M. Engdahl B. E. (1998). Polysomnographic sleep is not clinically impaired in Vietnam combat veterans with chronic posttraumatic stress disorder. 44 1066–1073. 10.1016/S0006-3223(98)00089-4 9821572

[B21] Johnson L. C. Lubin A. (1966). Spontaneous electrodermal activity during waking and sleeping. 3 8–17. 10.1111/j.1469-8986.1966.tb02673.x 5942878

[B22] Karalis K. Muglia L. J. Bae D. Hilderbrand H. Majzoub J. A. (1997). Crh and the immune system. 72 131–136. 10.1016/S0165-5728(96)00178-6 9042104

[B23] Keshavan M. S. Prasad K. M. Montrose D. M. Miewald J. M. Kupfer D. J. (2007). Sleep quality and architecture in quetiapine, risperidone, or never-treated schizophrenia patients. 27 703–705. 10.1097/jcp.0b013e31815a884d 18004141

[B24] Kleinlogel H. (1989). Effects of the selective alpha 1-adrenoceptor blocker prazosin on eeg sleep and waking stages in the rat. 21 100–103. 10.1159/000118560 2559357

[B25] Kluge M. Schussler P. Steiger A. (2007). Duloxetine increases stage 3 sleep and suppresses rapid eye movement (rem) sleep in patients with major depression. 17 527–531. 10.1016/j.euroneuro.2007.01.006 17337164

[B26] Kobayashi I. Boarts J. M. Delahanty D. L. (2007). Polysomnographically measured sleep abnormalities in ptsd: a meta-analytic review. 44 660–669. 10.1111/j.1469-8986.2007.537.x 17521374

[B27] Kushniruk A. Rustenburg J. Ogilvie R. (1985). Psychological correlates of electrodermal activity during rem sleep. 8 146–154. 10.1093/sleep/8.2.146 4012157

[B28] Lamarche L. J. De Koninck J. (2007). Sleep disturbance in adults with posttraumatic stress disorder: a review. 68 1257–1270. 10.4088/JCP.v68n0813 17854251

[B29] Lester B. K. Burch N. R. Dossett R. C. (1967). Nocturnal eeg-gsr profiles: The influence of presleep states. 3 238–248. 10.1111/j.1469-8986.1967.tb02701.x 6038667

[B30] Makela J. P. Hilakivi I. T. (1986). Effect of alpha-adrenoceptor blockade on sleep and wakefulness in the rat. 24 613–616. 10.1016/0091-3057(86)90566-6 2871563

[B31] Mellman T. A. Kumar A. Kulick-Bell R. Kumar M. Nolan B. (1995). Nocturnal/daytime urine noradrenergic measures and sleep in combat-related ptsd. 38 174–179. 10.1016/0006-3223(94)00238-X 7578660

[B32] Monti J. M. Torterolo P. Pandi Perumal S. R. (2017). The effects of second generation antipsychotic drugs on sleep variables in healthy subjects and patients with schizophrenia. 33 51–57. 10.1016/j.smrv.2016.05.002 27321864

[B33] Onton J. A. Kang D. Y. Coleman T. P. (2016). Visualization of whole-night sleep eeg from 2-channel mobile recording device reveals distinct deep sleep stages with differential electrodermal activity. 10:605. 10.3389/fnhum.2016.00605 27965558 PMC5126123

[B34] Pellejero T. Monti J. M. Baglietto J. Jantos H. Pazos S. Cichevski V. (1984). Effects of methoxamine and alpha-adrenoceptor antagonists, prazosin and yohimbine, on the sleep-wake cycle of the rat. 7 365–372. 10.1093/sleep/7.4.365 6515252

[B35] Peskind E. R. Bonner L. T. Hoff D. J. Raskind M. A. (2003). Prazosin reduces trauma-related nightmares in older men with chronic posttraumatic stress disorder. 16 165–171. 10.1177/0891988703256050 12967060

[B36] Raskind M. A. Peskind E. R. Hoff D. J. Hart K. L. Holmes H. A. Warren D. (2007). A parallel group placebo controlled study of prazosin for trauma nightmares and sleep disturbance in combat veterans with post-traumatic stress disorder. 61 928–934. 10.1016/j.biopsych.2006.06.032 17069768

[B37] Rechtschaffen A. Kales A. (1968). *A Manual of Standardized Terminology, Techniques and Scoring System for Sleep Stages of Human Subjects*. Washington, DC: US Government Printing Office.

[B38] Salin-Pascual R. J. Galicia-Polo L. Drucker-Colin R. (1997). Sleep changes after 4 consecutive days of venlafaxine administration in normal volunteers. 58 348–350. 10.4088/JCP.v58n0803 9515972

[B39] Salin-Pascual R. J. Moro-Lopez M. L. (1997). Effects of venlafaxine in the sleep architecture of rats. 129 295–296. 10.1007/s002130050194 9084070

[B40] Sanchez C. Brennum L. T. Storustovu S. Kreilgard M. Mork A. (2007). Depression and poor sleep: the effect of monoaminergic antidepressants in a pre-clinical model in rats. 86 468–476. 10.1016/j.pbb.2007.01.006 17303232

[B41] Sano A. Picard R. W. Stickgold R. (2014). Quantitative analysis of wrist electrodermal activity during sleep. 94 382–389. 10.1016/j.ijpsycho.2014.09.011 25286449 PMC4335672

[B42] Sequeira H. Hot P. Silvert L. Delplanque S. (2009). Electrical autonomic correlates of emotion. 71 50–56. 10.1016/j.ijpsycho.2008.07.009 18723054

[B43] Sharpley A. L. Williamson D. J. Attenburrow M. E. Pearson G. Sargent P. Cowen P. J. (1996). The effects of paroxetine and nefazodone on sleep: a placebo controlled trial. 126 50–54. 10.1007/BF02246410 8853216

[B44] Shrivastava D. Jung S. Saadat M. Sirohi R. Crewson K. (2014). How to interpret the results of a sleep study. 4 10.3402/jchimp.v4.24983 PMC4246141 25432643

[B45] Steriade M. Nunez A. Amzica F. (1993). Intracellular analysis of relations between the slow (<1 hz) neocortical oscillation and other sleep rhythms of the electroencephalogram. 13 3266–3283. 10.1523/JNEUROSCI.13-08-03266.1993 PMC6576520 8340807

[B46] Taylor F. Raskind M. A. (2002). The alpha1-adrenergic antagonist prazosin improves sleep and nightmares in civilian trauma posttraumatic stress disorder. 22 82–85. 10.1097/00004714-200202000-00013 11799347

[B47] Taylor F. B. Martin P. Thompson C. Williams J. Mellman T. A. Gross C. (2008). Prazosin effects on objective sleep measures and clinical symptoms in civilian trauma posttraumatic stress disorder: a placebo-controlled study. 63 629–632. 10.1016/j.biopsych.2007.07.001 17868655 PMC2350188

[B48] Ulmer C. S. Edinger J. D. Calhoun P. S. (2011). A multi-component cognitive-behavioral intervention for sleep disturbance in veterans with ptsd: a pilot study. 7 57–68. 21344046 PMC3041620

[B49] von Kanel R. Hepp U. Kraemer B. Traber R. Keel M. Mica L. (2007). Evidence for low-grade systemic proinflammatory activity in patients with posttraumatic stress disorder. 41 744–752. 10.1016/j.jpsychires.2006.06.009 16901505

[B50] Woodward S. H. Bliwise D. L. Friedman M. J. Gusman D. F. (1996). Subjective versus objective sleep in Vietnam combat veterans hospitalized for ptsd. 9 137–143. 10.1002/jts.2490090112 8750457

[B51] Woodward S. H. Murburg M. M. Bliwise D. L. (2000). Ptsd-related hyperarousal assessed during sleep. 70 197–203. 10.1016/S0031-9384(00)00271-7 10978496

